# Impairment of Granzyme B-Producing Regulatory B Cells Correlates with Exacerbated Rheumatoid Arthritis

**DOI:** 10.3389/fimmu.2017.00768

**Published:** 2017-06-30

**Authors:** Liling Xu, Xu Liu, Hongjiang Liu, Lei Zhu, Huaqun Zhu, Jian Zhang, Limin Ren, Pingzhang Wang, Fanlei Hu, Yin Su

**Affiliations:** ^1^Department of Rheumatology and Immunology, Peking University People’s Hospital, Beijing, China; ^2^Beijing Key Laboratory for Rheumatism Mechanism and Immune Diagnosis (BZ0135), Beijing, China; ^3^Peking-Tsinghua Center for Life Sciences, Beijing, China; ^4^Department of Immunology, School of Basic Medical Science, Peking University, Beijing, China

**Keywords:** rheumatoid arthritis, granzyme B, regulatory B cells, impairment, T cells

## Abstract

Hyperactivated B cells have been demonstrated the contribution to the development of rheumatoid arthritis (RA). While the recognition of the negative regulatory function of B cells further promoted our understanding of their pathogenic role in RA. Recently, a new population of granzyme B (GrB)-producing B cells was identified, which was proved to be involved in cancer and infectious diseases. However, their characteristics and roles in RA remain to be elucidated. In the present study, we aim to further characterize whether B cells could produce GrB and reveal their potential role in the pathogenesis of RA. Here, we further demonstrated peripheral blood B cells from healthy individuals could produce and secrete GrB, which could be enhanced by IL-21 and/or anti-B-cell receptor stimulation. These cells could negatively regulate Th1 and Th17 cells partly *via* downregulating TCR zeta chain and inducing T cell apoptosis, which might be termed as GrB-producing regulatory B cells (Bregs). These GrB-producing Bregs were significantly decreased under RA circumstance concomitant of lower levels of IL-21 receptor, with impaired regulatory functions on Th1 and Th17 cells. Moreover, the frequencies of these cells were negatively correlated with RA patient disease activity and clinical features. After effective therapy with disease remission in RA, these GrB-producing Bregs could be recovered. Therefore, our data revealed that B cells could produce GrB with immunosuppressive functions, and the impairment of this Breg subset was correlated with RA pathogenesis.

## Introduction

Rheumatoid arthritis (RA) is a common and complex autoimmune disease characterized by chronic inflammation and cartilage/bone damage with dysregulation of various cells, such as T cells, B cells, chondrocytes, and fibroblasts (1). Although the precise etiology and pathogenesis of RA remain unclear, B cells are viewed as one of the most important participants in the initiation and perpetuation of RA (2, 3).

Hyperactivated B cells have long been thought to contribute to the development of RA since the discovery of rheumatoid factor (RF) and autoantibodies against citrullinated peptides (3). Furthermore, B cells are known to dominate immune activation through antigen presentation and cytokine secretion, which also play an important role in RA pathogenesis (4). Nevertheless, B cells have also been shown to play a significant role in the negative regulation of immune response recently, which is an important advance for promoting our better understanding of the role of B cells in RA (5). These regulatory B cells (Bregs), particularly IL-10-producing Breg (B10), were proved to be numerically decreased and negatively correlated with disease activity in RA (6). In addition, our studies further demonstrated that B10 cells not only lost their immunosuppressive functions but also pathogenically converted into RANKL-producing cells under RA circumstance (7).

Besides B10 cells, other Breg subsets were also identified, and their critical roles were demonstrated under physiologic and pathologic conditions (8–10). Recently, Lindner et al. revealed a new population of B cells that may play a regulatory role *via* the release of granzyme B (GrB). GrB is a member of the serine protease family mainly produced by cytotoxic cells like cytotoxic T lymphocytes and natural kill (NK) cells, which is traditionally considered to induce target cell apoptosis with perforin (11). Although most cell types express both GrB and perforin simultaneously, recent studies showed that GrB could be released by other cells independent of perforin (12–14), suggesting that GrB may act with extracellular activity (15). Lindner et al. also found that GrB-producing B cells could suppress the proliferation of CD4^+^ T cells by cleaving TCR zeta chain with GrB-dependent and perforin-independent manner (16). These GrB-producing B cells were proved to play an important role in cancer and virus infection *via* the release of GrB (16–18). However, the characteristics of GrB-producing B cells and their potential role in RA are largely unknown.

In this study, we further demonstrated that B cells could secrete GrB with negative regulation on Th1 and Th17 cells, which was partly mediated by downregulating TCR zeta chain and inducing T cell apoptosis. GrB-producing B cells were numerically and functionally impaired under RA circumstance, which were also correlated with patient disease activity. Therefore, our results further supported the existence of GrB-producing Breg in humans and might provide a new insight into the role of B cells in RA pathogenesis.

## Materials and Methods

### Patients and Controls

Patients with RA (*n* = 60) (Table 1) meeting 1987 American College of Rheumatology revised criteria (19) were enrolled from the Department of Rheumatology and Immunology, Peking University People’s Hospital, China. Clinical and laboratory parameters were collected. Disease activity was measured using disease activity score based on 28 joints (DAS28). Osteoarthritis (OA) patients (*n* = 15) meeting 1986 American College of Rheumatology criteria (20) with matched age and gender were recruited for disease control. 45 healthy individual samples were obtained from physical examination center in our hospital. All the samples used in this study were freshly collected from patients and healthy controls. The study was approved by Institutional Medical Ethics Review Board of Peking University People’s Hospital, and all the participants provided written informed consent.

**Table 1:** Clinical characteristics of the RA patients included in the study.

Characteristics	RA (*n* = 60)
Age, mean (range), years	57 (22–82)
Sex, no. female/male	54/6
Duration, mean (range), years	10.7 (0.17–40)
Tender joint count, median (range) of 28 joints	7 (0–28)
Swollen joint count, median (range) of 28 joints	4 (0–28)
DAS28, mean (range)	5.06 (1.21–7.93)
ESR, mean (range), mm/h	48 (2–114)
CRP, mean (range), mg/l	32.5 (1.42–165)
RF, mean (range), IU/ml	399 (20–2,600)
Anti-CCP antibody, mean (range), U/ml	203.4 (3.2–339.5)

*ESR, erythrocyte sedimentation rate; CRP, C-reactive protein; RF, rheumatoid factor; Anti-CCP antibody, anti-cyclic citrullinated peptide antibody; RA, rheumatoid arthritis*.

### Antibodies and Reagents

Phorbol 12-myristate 13-acetate (PMA), ionomycin, and Brefeldin A (BFA) were obtained from Multisciences (China). Recombinant human IL-21 (rhIL-21) was purchased from PeoproTech GmbH (Rocky Hill, CT, USA). Anti-GrB antibody and isotype control were purchased from R&D Systems (Minneapolis, MN, USA). CpG ODN 2006 was purchased from InvivoGen (San Diego, CA, USA). Anti-human IgM + IgG for B-cell receptor (BCR) stimulation and APC-Cyanine7-anti-human CD19 antibody, FITC-anti-human CD4 antibody, PerCP-Cyanine5.5-anti-human CD4 antibody, FITC-anti-human CD14 antibody, PE-anti-human-IL-21 receptor (IL-21R) antibody, PE-Cyanine7-anti-human CD25 antibody, PE-anti-human GrB antibody, FITC-anti-human IFN-gamma antibody, and eFluor^®^ 660-anti-human IL-17A antibody for immunostaining were purchased from eBioscience (San Diego, CA, USA). Alexa Fluor^®^ 700-anti-human CD3 antibody, Brilliant Violet 570-anti-human CD56 antibody, APC-annexin V, and 7-AAD viability staining solution were purchased from Biolegend (San Diego, CA, USA).

### Flow Cytometry

Peripheral blood mononuclear cells (PBMCs) were isolated from fresh heparinized venous blood samples using Ficoll density-gradient centrifugation. Before flow cytometry, single cell suspensions were incubated with CD16/CD32 Fc receptor blocker for 30 min.

For intracellular staining, cells were incubated with PMA (50 ng/ml), ionomycin (1 µg/ml), and BFA (10 µg/ml) for 5 h, then surface stained, fixed, permeabilized and intracellular stained in accordance with the manufacturer’s instructions. For intracellular GrB detection in B cells, dump channel staining with anti-CD3 antibody, anti-CD56 antibody, and anti-CD14 antibody was performed to exclude interference from other cell subsets. Dead cell exclusion was performed by scatter profiles and 7-ADD staining during all the flow cytometric analyses.

CD19^+^ B cells, CD4^+^CD25^−^ T cells, and CD8^+^ T cells were purified from PBMCs using FACS sorter by surface staining according to the manufacturer’s instructions. For B cell sorting, PBMCs were stained with anti-CD3 antibody, anti-CD56 antibody, anti-CD19 antibody, and anti-CD14 antibody to exclude interference from other cell subsets. And purified CD19^+^ B cells were further analyzed after sorting, the purity of which was 95–99%.

### PCR and Q-PCR Analysis of GrB Expression

Total RNA was isolated from purified CD19^+^ B cells using RNeasy mini kit (Qiagen, Hilden), then reverse transcribed into the oligo (dT)-primed cDNA by RevertAid FirstStrand kit (Fermentas, Glen Burnie, MD, USA). PCR and real-time quantitative PCR (Q-PCR) were performed to analyze the expression of GrB mRNA according to the manufacturer’s protocol. The sequences of the primers used in this study were as follows: the forward GAPDH primer was 5′-AAGGTGAAGGTCGGAGTCAA-3′ and the reverse GAPDH primer was 5′-AATGAAGGGGTCATTGATGG-3′; the forward GrB primer was 5′-TGCAGGAAGATCGAAAGTGCG-3′ and the reverse GrB primer was 5′-GAGGCATGCCATTGTTTCGTC-3′.

### GrB-Specific ELISpot Assay

*Ex vivo* GrB-ELISpot assays using purified CD19^+^ B cells were performed according to the manufacturer’s instructions (Mabtech, Sweden). CD19^+^ B cells from healthy individuals or RA patients were plated in RPMI 1640 medium (Life Technologies, Grand Island, NY, USA) supplemented with 10% FBS (Life Technologies) at 2.5 × 10^5^ cells per 200 µl per well under CpG (10 µg/ml) stimulation with or without rhIL-21 (50 ng/ml) and anti-BCR (10 µg/ml) stimulation for 24 h. CD8^+^ T cells were chosen as positive control while medium was used as negative control. Plates were read on ImmunoSpot Analyzer (Cellular Technology Ltd., Shaker Heights, OH, USA).

### Th1 Cell and Th17 Cell Differentiation

CD19^+^ B cells and CD4^+^CD25^−^ T cells from freshly isolated PBMCs were purified by flow cytometry sorting. The purity of sorted CD19^+^ B cells and CD4^+^CD25^−^ T cells used for experiments was about 95–99%. Then 5 × 10^5^ CD4^+^CD25^−^ T cells were cocultured with 2 × 10^5^ CD19^+^ B cells (2.5:1) in the presence of anti-GrB antibody (10 µg/ml) or isotype antibody (10 µg/ml) for 3 days under the stimulation of anti-CD3 antibody (3 µg/ml), anti-CD28 antibody (3 µg/ml), CpG (10 µg/ml), rhIL-21 (50 ng/ml), and anti-BCR (10 µg/ml). Cells were harvested for intracellular staining, as described previously.

### Statistical Analysis

SPSS 20.0 for Windows (SPSS Inc., Chicago, IL, USA) was used for statistical analysis. The differences between groups were performed by Student’s *t*-test, non-parametric Mann–Whitney rank sum test, Wilcoxon signed-rank test, or one-way ANOVA with *post hoc* Dunnett multiple-comparison test (as appropriate). Spearman’s correlation coefficient was applied to assess the correlations between two variables. *P* value < 0.05 was considered statistically significant.

## Results

### Production of GrB by B Cells in Human Peripheral Blood

**Figure 1:**
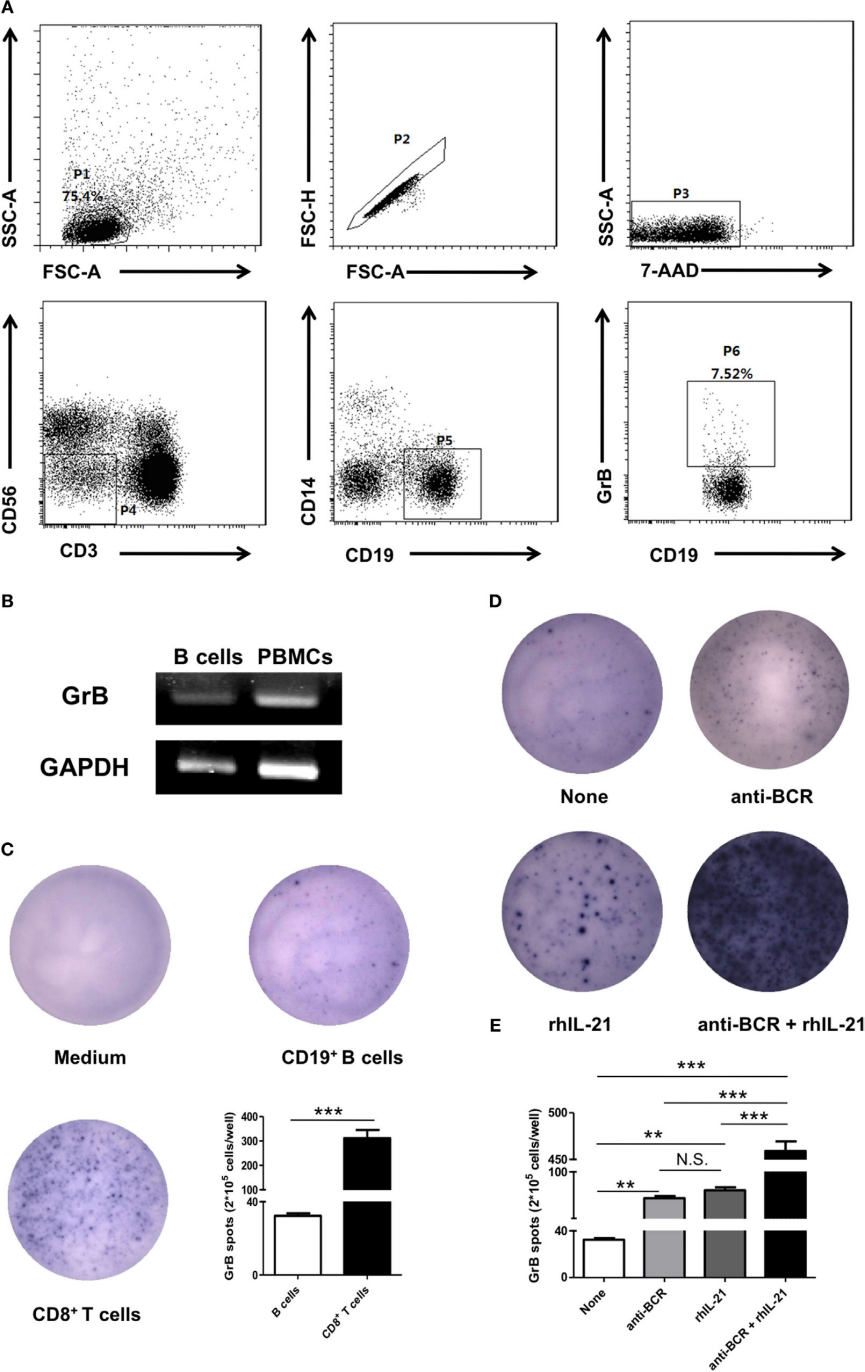
B cells in human peripheral blood produced and secreted GrB spontaneously. **(A)** Peripheral blood mononuclear cells were isolated from healthy individual fresh peripheral blood and incubated with phorbol 12-myristate 13-acetate (50 ng/ml), ionomycin (1 µg/ml), and Brefeldin A (10 µg/ml) for 5 h. Intracellular expression of GrB was detected in B cells by staining with anti-CD19 antibody, anti-CD3 antibody, anti-CD56 antibody, anti-CD14 antibody, and anti-GrB antibody. Dead cell exclusion was performed by scatter profiles and 7-ADD staining. FACS gating strategy and the representative scatter diagram from 15 healthy individuals were shown. **(B)** The GrB mRNA expression of purified CD19^+^ B cells (2 × 10^5^) was determined by PCR. The transcript of human GAPDH gene was used as an amplification control. The representative data of five independent experiments were shown. **(C)** 2 × 10^5^ freshly FACS-sorted CD19^+^ B cells (middle) from healthy individuals were cultured with CpG stimulation (10 µg/ml) on GrB-specific ELISpot plates for 24 h. Medium and CD8^+^ T cells were used as blank control and positive control, respectively, then dots were counted. Shown was representative of independent data from six different donors. **(D,E)** 2.5 × 10^5^ CD19^+^ B cells per well were cultured on GrB-specific 96-well ELISpot plates under CpG stimulation (10 µg/ml) with or without following stimuli: recombinant human IL-21 (rhIL-21) (50 ng/ml) and/or anti-B-cell receptor (BCR) (10 µg/ml) as indicated, then plates were developed for 24 h and dots were counted. One representative experiment out of five **(D)** and the statistical results were shown **(E)**. **P < 0.01, ***P < 0.001, N.S., no significance, by Student’s *t*-test, one-way ANOVA with *post hoc* Dunnett’s *t* test. GrB, granzyme B.

To determine whether human peripheral blood B cells could produce GrB, we firstly isolated PBMCs from 15 healthy individual fresh samples for further staining with anti-CD19 antibody, anti-CD3 antibody, anti-CD56 antibody, anti-CD14 antibody, and anti-GrB antibody, then analyzed by flow cytometry. It was found that human peripheral blood B cells (CD3^−^CD56^−^CD14^−^CD19^+^) showed a moderate potency in producing GrB (Figure 1A). To further validate our finding, we also verified the expression of GrB by PCR in FACS-sorted B cells (Figure 1B), the purity of which was confirmed by FACS (Figure S1 in Supplementary Material).

Granzyme B, a main member of serine proteinase superfamily, played a critical role with the releasing pattern of secretory granules in physiological and pathological conditions. To investigate whether GrB produced by B cells was actively secreted, we performed GrB-specific ELISpot analysis (Figure 1C). The results showed that comparing with CD8^+^ T cells, B cells could also secrete GrB at moderate levels. Collectively, these results suggested that human peripheral blood B cells demonstrated the ability to produce and secrete GrB spontaneously.

We next tested the potential regulators of GrB production in B cells. IL-21 as a novel member of IL-2 cytokine family had pleiotropic functions on multiple cells, such as NK cells and B cells. Meanwhile, considering that BCR is closely connected with the proliferation and differentiation of B cells, we addressed whether the production of GrB by B cells could be enhanced in the presence of IL-21 and/or anti-BCR stimulation. FACS-purified peripheral blood B cells were cultured on GrB-specific ELISpot plates and stimulated with or without IL-21 and anti-BCR for 24 h. We found the significant upregulation of B cell produced GrB under IL-21 or anti-BCR stimulation alone, which was further enhanced when these two stimuli were add simultaneously (Figures 1D,E).

### Negative Regulation of CD4^+^ Effector T Cells by GrB-Producing B Cells

**Figure 2:**
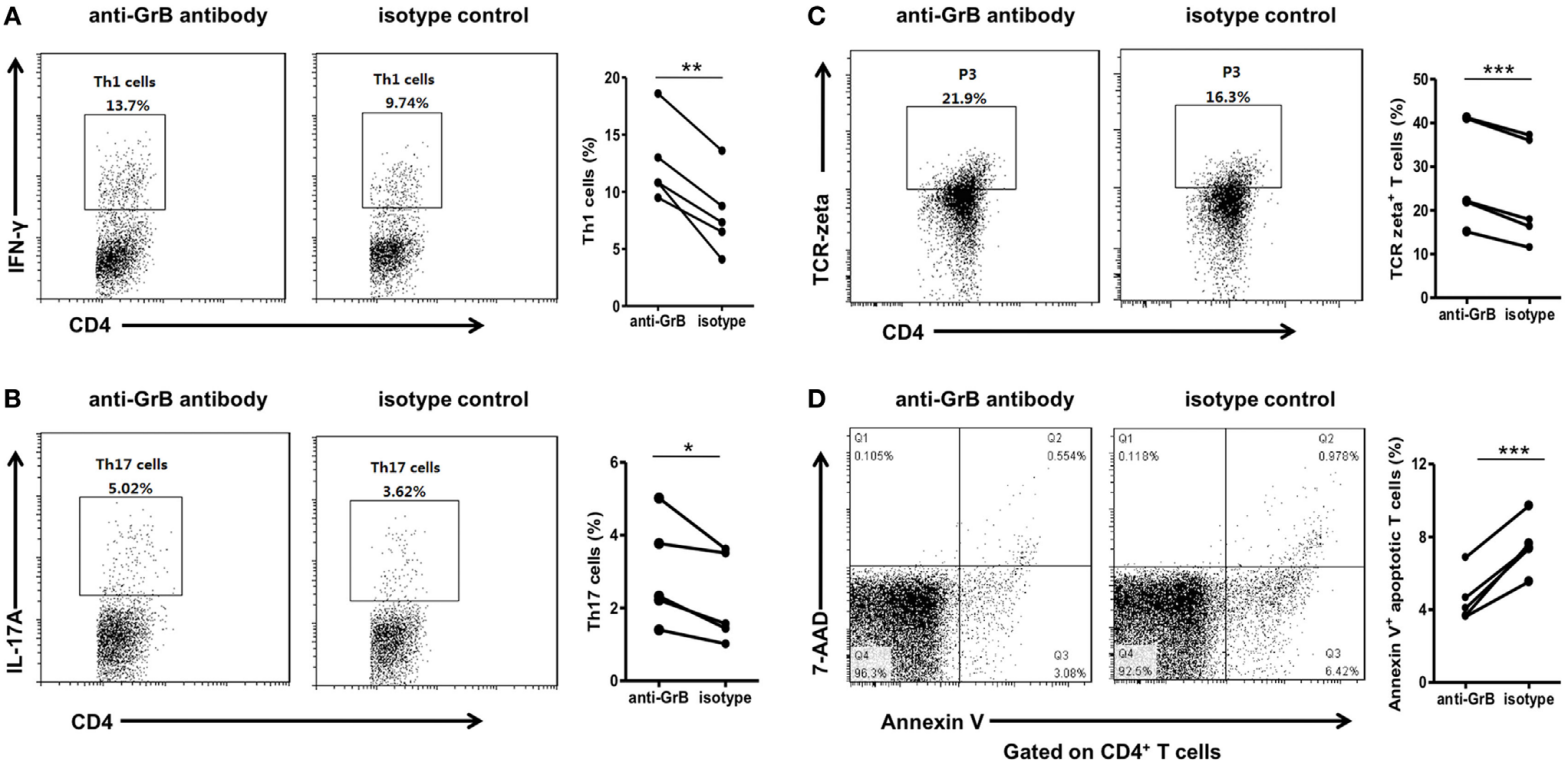
GrB-producing B cells from healthy individuals inhibited Th1 and Th17 cell responses. Purified peripheral blood CD19^+^ B cells and CD4^+^CD25^−^ T cells from healthy individuals (*n* = 10) were cocultured with anti-CD3 antibody (3 µg/ml), anti-CD28 antibody (3 µg/ml), CpG stimulation (10 µg/ml), recombinant human IL-21 (50 ng/ml), and anti-B-cell receptor (10 µg/ml) in the presence of anti-GrB antibody or isotype control. After 3 days, the cells were stained with anti-CD4 antibody, anti-IFN-gamma antibody, anti-IL-17A antibody, anti-TCR-zeta antibody, annexin V, and 7-AAD and analyzed by FACS. **(A)** The representative frequencies of Th1 cells measured by FACS were showed, which were obviously increased in the presence of anti-GrB antibody. **(B)** The frequencies of Th17 cells under the blockade of GrB were detected by FACS. The frequencies of TCR zeta^+^ T cells **(C)** and apoptotic T cells **(D)** were detected by flow cytometry and the representative flowcharts as well as the statistical results were shown. *P < 0.05, **P < 0.01, ***P < 0.001, by Wilcoxon signed-rank test. GrB, granzyme B.

We then analyzed the function of GrB-producing B cells. It has been demonstrated that B cells were able to negatively regulate cellular immunity by producing immunoregulatory cytokines, such as interleukin-10. And some research suggested an important GrB-mediated suppression role of GrB-producing regulatory T cells in keeping tumor tolerance and transplant. However, the effects of GrB-producing B cells on CD4^+^ effector T cell were still unclear. Therefore, we further co-incubated peripheral blood purified CD19^+^ B cells and CD4^+^ CD25^−^ T cells from healthy individuals in the presence of anti-GrB antibody or isotype control for 3 days, then collected the cells for Th1 and Th17 detection by flow cytometry. It was found that the frequencies of Th1 and Th17 cells were significantly elevated under the condition of GrB blockade (Figures 2A,B).

To further explore the possible mechanism, we detected the expression of TCR zeta and T cell apoptosis when CD4^+^CD25^−^ T cells and B cells were co-incubated with anti-GrB antibody or isotype antibody. We found that T cells were inhibited by the downregulation of TCR zeta chain, as demonstrated by flow cytometry analysis, which was consistent with Lindner et al. (Figure 2C). Moreover, the frequencies of apoptotic T cells were weakly but significantly decreased when GrB was neutralized (Figure 2D). All these results indicated that GrB-producing B cells could serve as a new B cell subset that may also had immunosuppressive functions. These cells might be termed as GrB-producing Bregs.

### Dimission of GrB-Producing Breg in RA Patients

**Figure 3:**
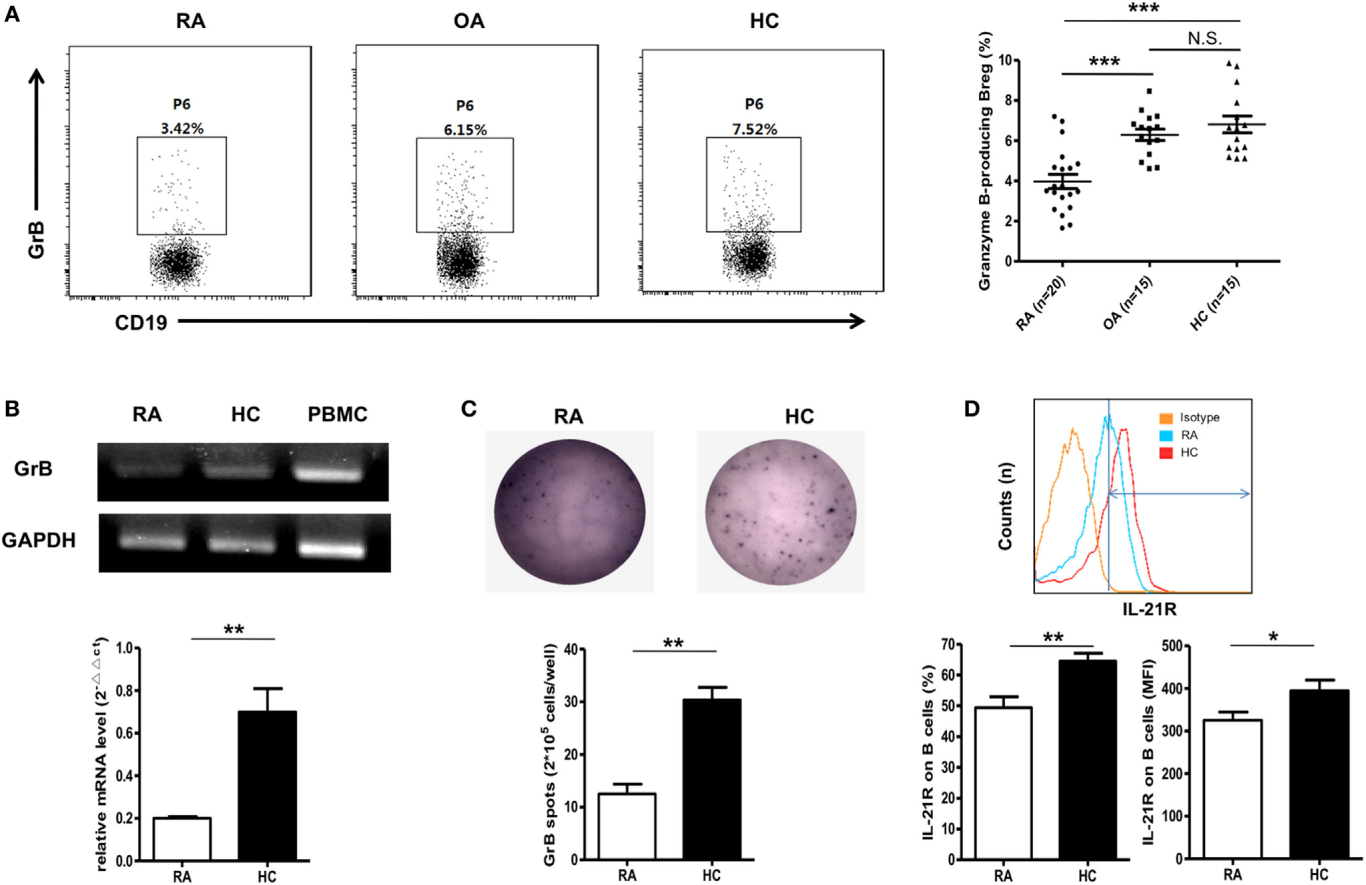
Reduction of GrB-producing regulatory B cell (Breg) in rheumatoid arthritis (RA) patients. **(A)** The frequencies of GrB-producing Breg were assayed by FACS in RA patients (*n* = 20), osteoarthritis (OA) patients (*n* = 15), and healthy donors (*n* = 15) as in Figure 1A. The representative flowcharts (left) and statistical results were showed (right) (one-way ANOVA with *post hoc* Dunnett’s *t* test). **(B)** Purified CD19^+^ B cells by FACS from five RA patients and five healthy controls were subjected to detection of mRNA expression of GrB by PCR (upper) and Q-PCR (bottom). **(C)** B cells from four RA patients and four healthy controls were cultured with CpG stimulation (10 µg/ml) on ELISpot plates at the density of 2.5 × 10^5^ cells/well for 24 h. Plates were developed, and GrB-secreting cells were counted. The representative photos (upper) and statistical results (bottom) were shown. **(D)** The frequencies and median fluorescent intensity of IL-21 receptor (IL-21R) on B cells were assayed by FACS in RA patients (*n* = 15) and healthy donors (*n* = 20). The represent flowcharts and statistical results were shown. *P < 0.05, **P < 0.01, ***P < 0.001, N.S., no significance, by Mann–Whitney test. GrB, granzyme B.

Next, we detected the frequencies of these GrB-producing Bregs under RA circumstance. 20 RA patients were included in this study, and 15 OA patients, 15 healthy individuals were chosen as the controls. Interestingly, as shown in Figure 3A, the frequencies of GrB-producing Breg were found to be obviously decreased in RA patients, while no significant difference was found between OA patients and healthy controls. PCR, Q-PCR, and ELISpot analysis further confirmed this dampened GrB-producing capacity of B cells in RA patients (Figures 3B,C).

As mentioned above, the frequencies of GrB-producing Breg were enhanced *in vitro* by stimulation with IL-21 engagement. Therefore, to address the question why B cells from RA patients exhibited lower expression of GrB, we performed the analysis of the levels of IL-21R on B cell membrane surface with FACS. The results showed that the expression of IL21R on B cells was obviously decreased in RA patients in comparison with healthy controls, which may be a possible mechanism for the reduction of GrB-producing Breg in RA patients (Figure 3D).

### Correlation Analysis of GrB-Producing Breg with RA Patient Clinical Features

**Figure 4:**
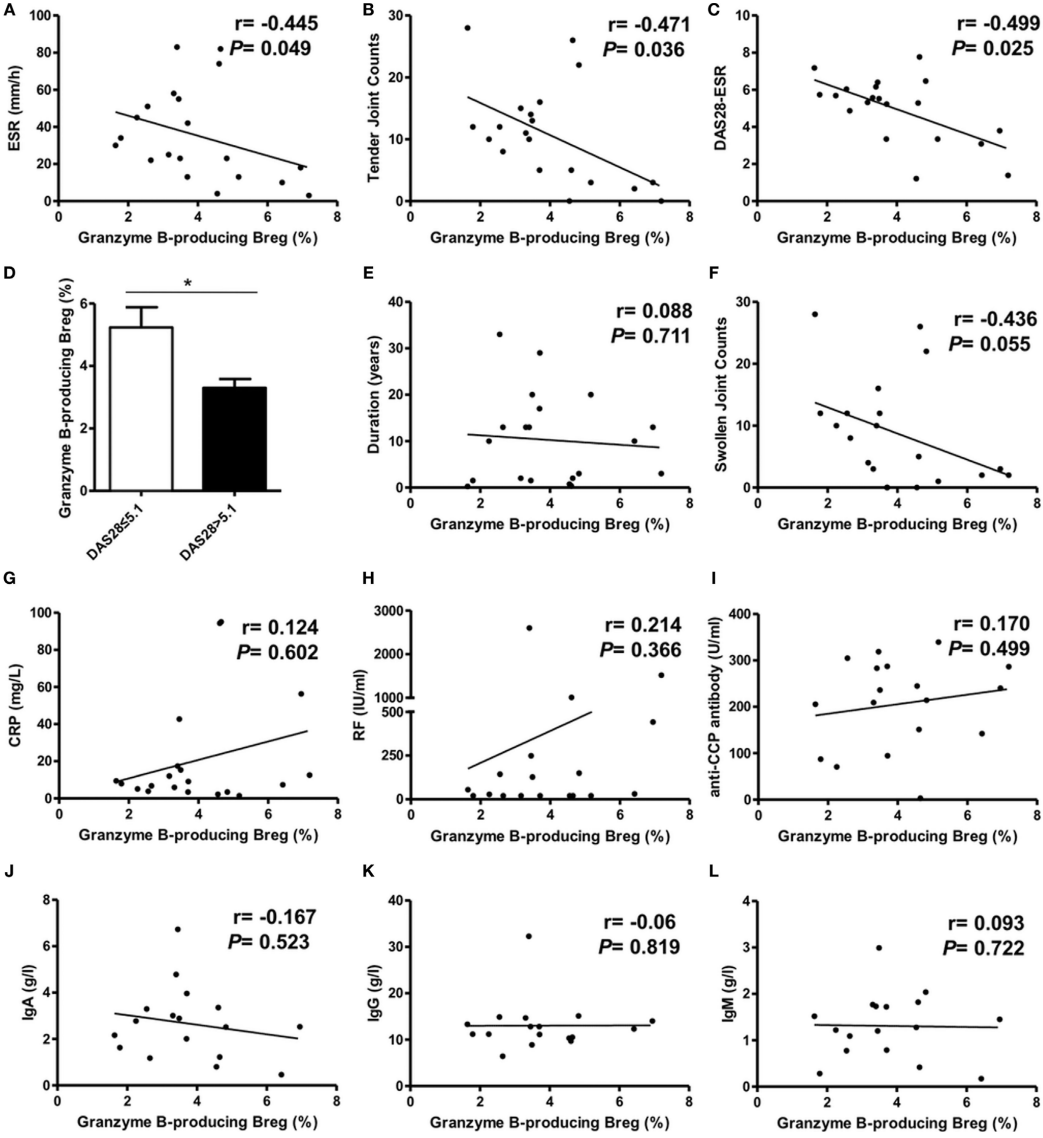
Correlation analysis of granzyme B (GrB)-producing regulatory B cell (Breg) with rheumatoid arthritis (RA) patient clinical manifestations. The frequencies of GrB-producing Breg were negatively correlated with RA patient ESR **(A)**, tender joint counts **(B)**, and DAS28-ESR **(C)**. The frequency difference of GrB-producing Breg was also analyzed between RA patients with high disease activity (DAS28 > 5.1) and non-high disease activity (DAS28 ≤ 5.1) **(D)**. The correlation of GrB-producing Breg with RA patient swollen joint counts **(E)**, CRP **(F)**, disease duration **(G)**, rheumatoid factor (RF) **(H)**, anti-CCP antibody **(I)**, serum IgA **(J)**, IgG **(K)**, and IgM **(L)** was also analyzed. *P < 0.05, by Spearman’s rank correlation test and Mann–Whitney test.

Next, we performed the correlation analysis between GrB-producing Breg and clinical features in RA patients. The results revealed a negative correlation between the frequencies of GrB-producing Breg and RA patient ESR, tender joint counts, and disease activity score DAS28 (Figures 4A–C). RA patients with high disease activity (DAS28 > 5.1) showed lower frequencies of GrB-producing Breg than those with non-high disease activity (DAS28 ≤ 5.1) (Figure 4D). No correlation was found between GrB-producing Breg and RA patient disease duration, swollen joint counts, the levels of CRP, RF, anti-CCP antibody, or serum IgA, IgG, and IgM (Figures 4E–L). Collectively, these results indicated that GrB-producing Breg, negatively correlating with the disease activity, might play an important role in the development of RA.

### Impairment of GrB-Producing Breg on Suppressing CD4^+^ Effector T Cell in RA Patients

**Figure 5:**
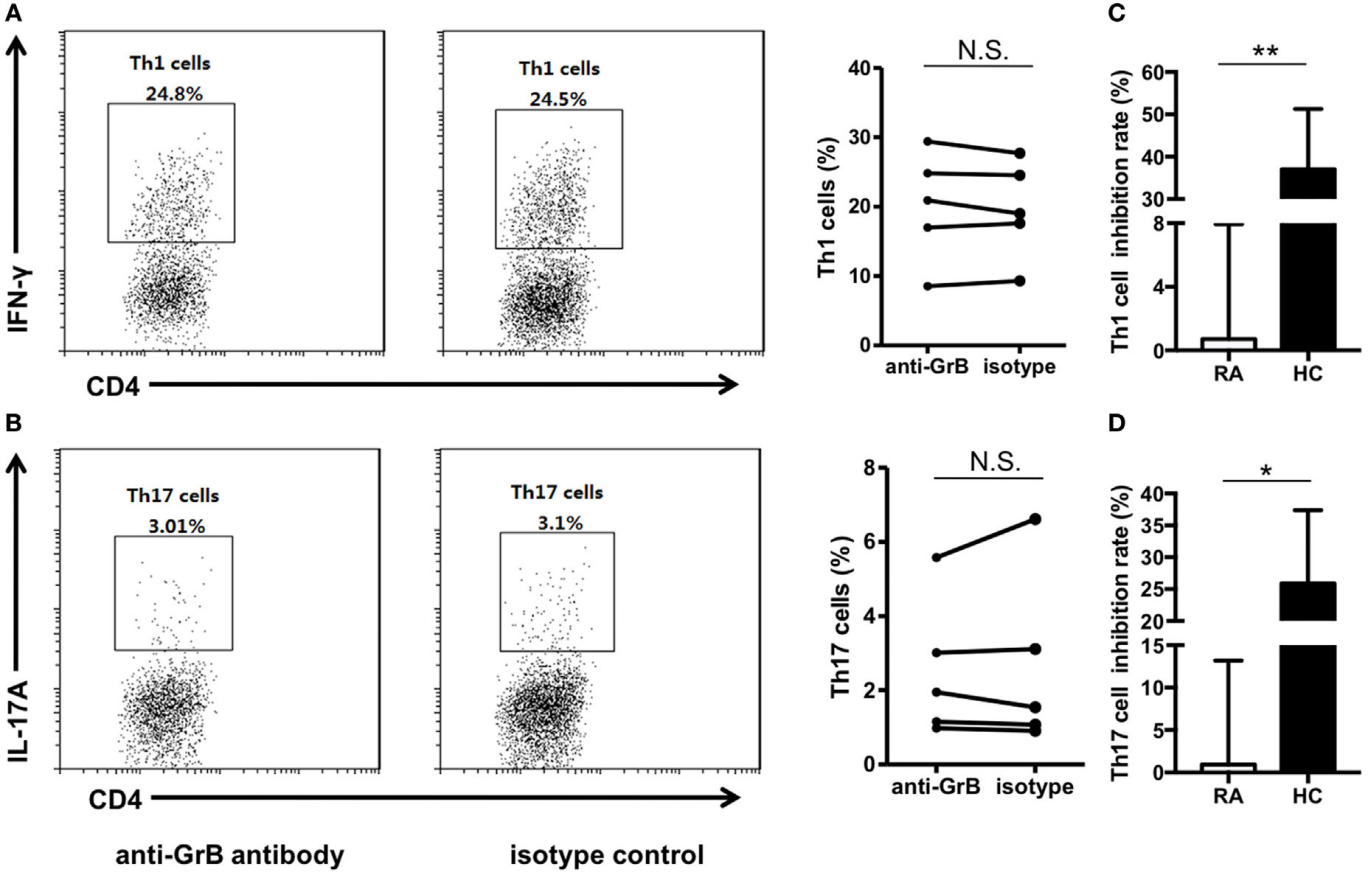
GrB-producing regulatory B cell (Breg) failed to suppress Th1 and Th17 cell responses in rheumatoid arthritis (RA) patients. **(A,B)** Peripheral blood CD19^+^ B cells and CD4^+^CD25^−^ T cells from RA patients (*n* = 5) were sorted and cocultured in the presence of anti-GrB antibody or isotype control as in Figure 2. The representative frequencies of Th1 **(A)** and Th17 **(B)** cells measured by FACS were showed, which demonstrated no significant difference between anti-GrB antibody group and isotype group. **(C,D)** The inhibition rates of GrB-producing Breg against Th1 **(C)** and Th17 **(D)** cells both in healthy controls and RA patients were calculated by the following formula: $\frac{\text{Th}1\text{ or Th}17{\text{ frequencies}}_{\text{anti-GrB group}}-\text{Th}1\text{ or Th}17{\text{ frequencies}}_{\text{isotype group}}}{\text{Th}1\text{ or Th}17{\text{ frequencies}}_{\text{anti-GrB group}}}\times 100\%$. *P < 0.05, **P < 0.01, N.S., no significance, by Mann–Whitney test. GrB, granzyme B.

We further analyzed the function of GrB-producing Breg in RA patients. FACS-sorted CD19^+^ B cells and CD4^+^CD25^−^ T cells isolated from RA patient peripheral blood samples were cocultured together in the presence of anti-GrB antibody or isotype control. The results showed that blockade of GrB activity showed no influence on the frequencies of Th1 and Th17 cells (Figures 5A,B), indicating the impaired suppressive functions of GrB-producing Breg on regulating CD4^+^ effector T cell-mediated inflammation in RA. Moreover, the inhibition rate analyses of both healthy control and RA patient GrB-producing Breg further confirmed this functional impairment (Figures 5C,D).

### Recovery of GrB-Producing Breg in RA Patients after Disease Remission

**Figure 6:**
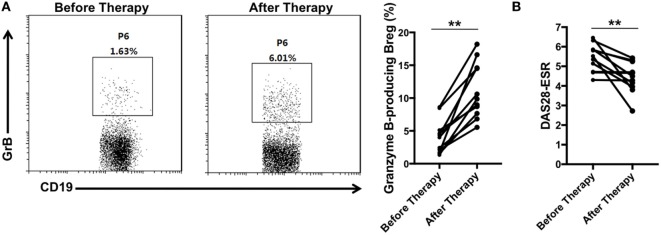
GrB-producing regulatory B cells (Bregs) were increased in rheumatoid arthritis (RA) patients with disease remission after therapy. 11 RA patients before and after therapy with disease-modifying antirheumatic drugs were enrolled in the study. **(A)** The frequencies of GrB-producing Breg were assayed by flow cytometry. The representative flowcharts and statistical results were shown. **(B)** The disease activity scores 28 (DAS28-ESR) of RA patients before and after therapy were evaluated and analyzed. **P < 0.01, by Wilcoxon signed-rank test. GrB, granzyme B.

Since GrB-producing Breg were decreased in RA patients and negatively correlated with the disease activity, we further evaluated whether these cells would be increased after effective therapy. With assessing the disease activity score DAS28 simultaneously, blood samples were collected from 11 RA patients before and after receiving therapy with disease-modifying antirheumatic drugs. We found that as the disease activity score DAS28 declined (Figure 6B), the frequencies of GrB-producing Breg were increased significantly (Figure 6A), indicating the recovery of these Bregs after disease remission. All these results suggested that the deficiency of GrB-producing Breg may be correlated with the development of RA.

## Discussion

In the present study, we further demonstrated that B cells could spontaneously produce GrB, which could be perpetuated by IL-21 and anti-BCR stimulation. These cells could negatively regulate Th1 and Th17 cell responses probably by downregulating TCR zeta chain and inducing T cell apoptosis, which might be termed as GrB-producing Breg. In RA patients, these GrB-producing Bregs were numerically decreased and functionally impaired, correlating with the patient clinical features. Moreover, after effective therapy, these GrB-producing Breg could get recovered.

Regulatory B cells have been demonstrated to contribute to the maintenance of immunologic equilibrium through inhibiting proinflammatory cytokine production and T-cell differentiation (21). In 2002, IL-10-producing B cells were identified and termed as regulatory B10 cells, which were the most extensively studied Breg subset (22). Besides B10, different Breg subsets were described, and the family of Breg was extending. Tian et al. and Parekh et al. identified the transforming growth factor β-producing capacity of B cells, which could induce the apoptosis of CD4^+^ effector T cells and the anergy of CD8^+^ T cells (9, 23). Shen et al. and Wang et al. reported IL-35-producing B cells as a critical regulator of immunity during infectious and autoimmune diseases (8, 24). Recently, some studies had elucidated the role of extracellular GrB, a member of serine protease family, with a non-apoptotic role in limiting lymphocytic inflammation and CD8^+^ T cell response (12, 25). Lindner et al. firstly revealed a new population of B cells that may play a regulatory role *via* the release of GrB, which was named as GrB-producing B cells (16). In the present study, we further proved that B cells had the capacity of producing GrB with regulatory functions on Th1 and Th17 cells, which may further improve our better understanding of Breg family.

However, the present way to identify GrB-producing Breg is intracellular cytokine staining by flow cytometry, which is due to the indeterminacy of the phenotype for these cells. Lindner et al. first reported the cell surface markers of GrB-producing Breg might be CD19^+^CD38^+^CD1d^+^IgM^+^CD147^+^ (16), while the following separate studies supported several different markers, such as CD38^+^CD20^−^ (26), CD19^+^IgG^+^IgD^−^CD27^−^ (27), or CD138^+^CD27^+^CD5^+^CD38^+^IgD^−^ (28). We also did some studies on their phenotype by bioinformatics analysis, which indicated that Fc fragment of IgE receptor Ig (FCER1G), CX3C chemokine receptor 1, TYRO protein tyrosine kinase binding protein (TYROBP), CD36, formyl peptide receptor 1, and IL7R might be their surface markers (data not shown), providing the potential reference for future study. Taken together, these results were intriguing, and further studies are still needed to identify the unique surface markers for this new Breg subset.

Regulatory B cells, particularly B10 cells have a variety of regulatory effects dependent on IL-10, such as inhibition of T cell activation, suppression of Th1 cell differentiation and maintenance of Treg pool (21). Previous study by Gondek and his colleagues indicated that GrB is one of the key mechanisms for CD4^+^CD25^+^ Treg-induced cell contact-mediated suppression (29). In this study, our results further showed that GrB-producing Breg also had a negative regulatory role on Th1 and Th17 cells with a GrB-dependent manner. The proportions of Th1 and Th17 cells were dramatically elevated under the condition of GrB blockade during CD4^+^ effector T cell-B cell coculture, which may be correlated with both the T cell apoptosis and the cleavage of TCR zeta chain in GrB-dependent manner.

Studies showed that Breg impairment contributed to the development of autoimmune diseases, including RA. Both our unpublished data and other groups’ studies proved that regulatory B10 cells were decreased and inversely correlated with disease activity under RA circumstance (6). Moreover, these B10 cells could pathogenically convert into RANKL-producing cells, thus promoting RA disease progression (7). Consistently, in this study we also found that in RA patients, but not OA patients, the frequencies of GrB-producing Breg, a new Breg subset, were decreased dramatically and negatively correlated with the disease activity score DAS28, which could recover to normal levels after effective treatment. More important, the regulatory roles of these cells on CD4^+^ effector T cell were impaired in RA patients. Thus, we proved for the first time that impairment of GrB-producing Breg may be correlated with RA incidence. However, the detail mechanisms and the potential pathogenic conversion of these cells need to be further studied.

## Ethics Statement

This study was carried out in accordance with the recommendations of “Institutional Medical Ethics Review Board of Peking University People’s Hospital” with written informed consent from all subjects. All subjects gave written informed consent in accordance with the Declaration of Helsinki. The protocol was approved by the “Institutional Medical Ethics Review Board of Peking University People’s Hospital.”

## Author Contributions

LX carried out most of the experiments and manuscript preparation. FH and YS conceived the study, participated in the design and interpretation of results, and reviewed and edited the manuscript. XL, HL, JZ, and LR helped to collect samples and clinical data. LZ and HZ participated in the experiments and data analysis. PW participated in the bioinformatics analysis.

## Conflict of Interest Statement

The authors declare that the research was conducted in the absence of any commercial or financial relationships that could be construed as a potential conflict of interest.
