# Supplementary material for: Impairment of Granzyme B-Producing Regulatory B Cells Correlates with Exacerbated Rheumatoid Arthritis
Source: Front Immunol. 2017 Jun 30;8:768. doi: 10.3389/fimmu.2017.00768 (PMC5491972; doi:10.3389/fimmu.2017.00768)
Supplement: Supplementary file 1 [file image_1.pdf]

# Supplementary Material:

## Impairment of granzyme B-producing regulatory B cells correlates with exacerbated rheumatoid arthritis

**Liling Xu, Xu Liu, Hongjiang Liu, Lei Zhu, Huaqun Zhu, Jian Zhang, Limin Ren, Pingzhang Wang, Fanlei Hu*, Yin Su***

* Correspondence:
Fanlei Hu: fanleihu@bjmu.edu.cn
Yin Su: suyin0921@163.com

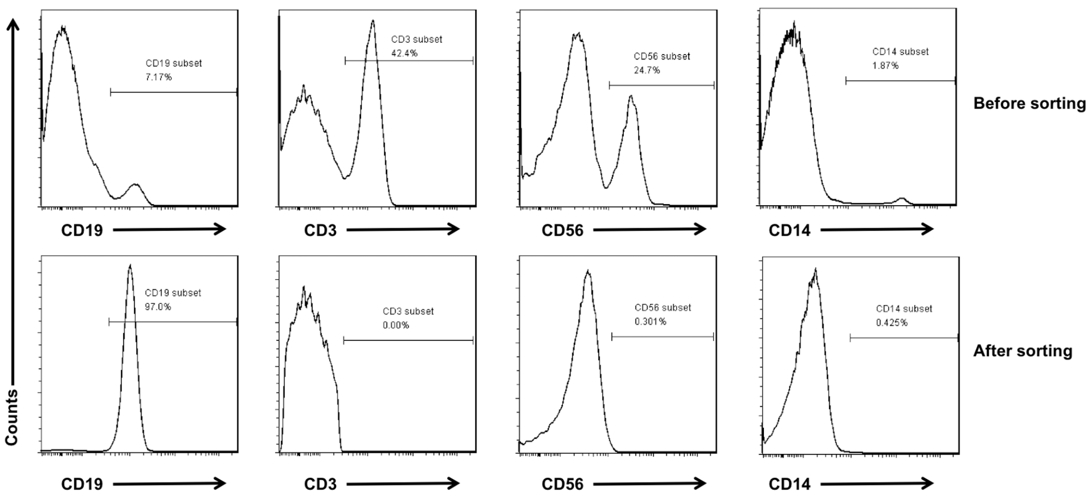


**Figure S1.** The purity analysis of FACS-sorted B cells. PBMCs were stained with anti-CD3 antibody, anti-CD56 antibody, anti-CD19 antibody and anti-CD14 antibody to exclude interference from other cell subsets, then the purity of the sorted cells was analyzed.
